# Caspase-4 and -5 Biology in the Pathogenesis of Inflammatory Bowel Disease

**DOI:** 10.3389/fphar.2022.919567

**Published:** 2022-05-31

**Authors:** Aoife P. Smith, Emma M. Creagh

**Affiliations:** School of Biochemistry and Immunology, Trinity Biomedical Sciences Institute, Trinity College Dublin, Dublin, Ireland

**Keywords:** inflammatory bowel disease, caspase-4, caspase-5, caspase-11, non-canonical inflammasome, pyroptosis

## Abstract

Inflammatory bowel disease (IBD) is a chronic relapsing inflammatory disease of the gastrointestinal tract, associated with high levels of inflammatory cytokine production. Human caspases-4 and -5, and their murine ortholog caspase-11, are essential components of the innate immune pathway, capable of sensing and responding to intracellular lipopolysaccharide (LPS), a component of Gram-negative bacteria. Following their activation by LPS, these caspases initiate potent inflammation by causing pyroptosis, a lytic form of cell death. While this pathway is essential for host defence against bacterial infection, it is also negatively associated with inflammatory pathologies. Caspases-4/-5/-11 display increased intestinal expression during IBD and have been implicated in chronic IBD inflammation. This review discusses the current literature in this area, identifying links between inflammatory caspase activity and IBD in both human and murine models. Differences in the expression and functions of caspases-4, -5 and -11 are discussed, in addition to mechanisms of their activation, function and regulation, and how these mechanisms may contribute to the pathogenesis of IBD.

## Introduction

Inflammatory bowel disease (IBD) is an idiopathic disorder characterised by chronic relapsing inflammation of the gastrointestinal (GI) tract. IBD presents in two main forms, namely Crohn’s disease (CD) and ulcerative colitis (UC) (Guan, 2019). IBD represents one of the most prevalent GI disorders, its incidence markedly rising during the second half of the 20th century and the beginning of the 21st century (Hanauer, 2006; Ng et al., 2017). Prevalence of IBD is highest in Europe and Northern America, however, recently incidence rates have remained stable or even declined. In contrast, incidence is increasing in newly industrialised countries, such as South America, Asia, and Africa highlighting the impact of environmental factors on IBD (Ng et al., 2017).

GI inflammation in IBD patients results in clinical symptoms including abdominal pain, diarrhoea, bloody stools, and weight loss (Guan, 2019). Transmural inflammation occurring non-continuously anywhere throughout the GI tract associated with complications such as strictures, fistulas, and granulomas is indicative of CD. While inflammation restricted to the mucosal layer of the colon and rectum associated with cryptitis and crypt abscesses is suggestive of UC (Hanauer, 2006; Abraham and Cho, 2009). The exact pathogenesis of IBD remains elusive, however, it is clearly multifactorial and influenced by both genetic as well as environmental factors. Overall, IBD can be described as a loss of immune homeostasis within the GI tract resulting in loss of barrier function, infiltration of immune cells into the lamina propria and high production of inflammatory cytokines (Guan, 2019).

Intestinal immunity is divided into innate and adaptive responses. The innate immune response is immediate and non-specific while the adaptative response requires prior innate immune priming and is highly specific (Yuan and Walker, 2004). The GI tract plays a pivotal role in maintaining innate immunity by providing a selective barrier which protects the host against pathogenic microorganisms while also inducing tolerance to food antigens and commensal bacteria, which form the microbiome (Guan, 2019). In order to appropriately initiate either a defensive or a tolerogenic response, immune signalling within the GI tract must be tightly regulated. Expression of germline encoded sensors termed pattern recognition receptors (PRRs) by intestinal epithelial cells (IECs) and innate immune cells within the GI tract aid in discriminating between harmful and harmless antigens (Yuan and Walker, 2004; Guan, 2019). PRRs bind pathogen-associated molecular patterns (PAMPs) and damage associated molecular patterns (DAMPs). PAMPs refer to a group of molecular patterns conserved among multiple pathogens that are necessary for pathogen survival. Lipopolysaccharide (LPS), a major component of the outer membrane of Gram-negative bacteria, is an example of a prototypical PAMP. In contrast to PAMPs, which are derived from exogenous pathogens, DAMPs refer to endogenously derived molecular patterns that signal cell damage or stress. These typically take the form of intercellular molecules such as, adenosine triphosphate (ATP) and high-mobility group box 1 (HMGB1), as their presence in the extracellular space signals that cell damage or stress has occurred (Boyapati et al., 2016).

**FIGURE 1:**
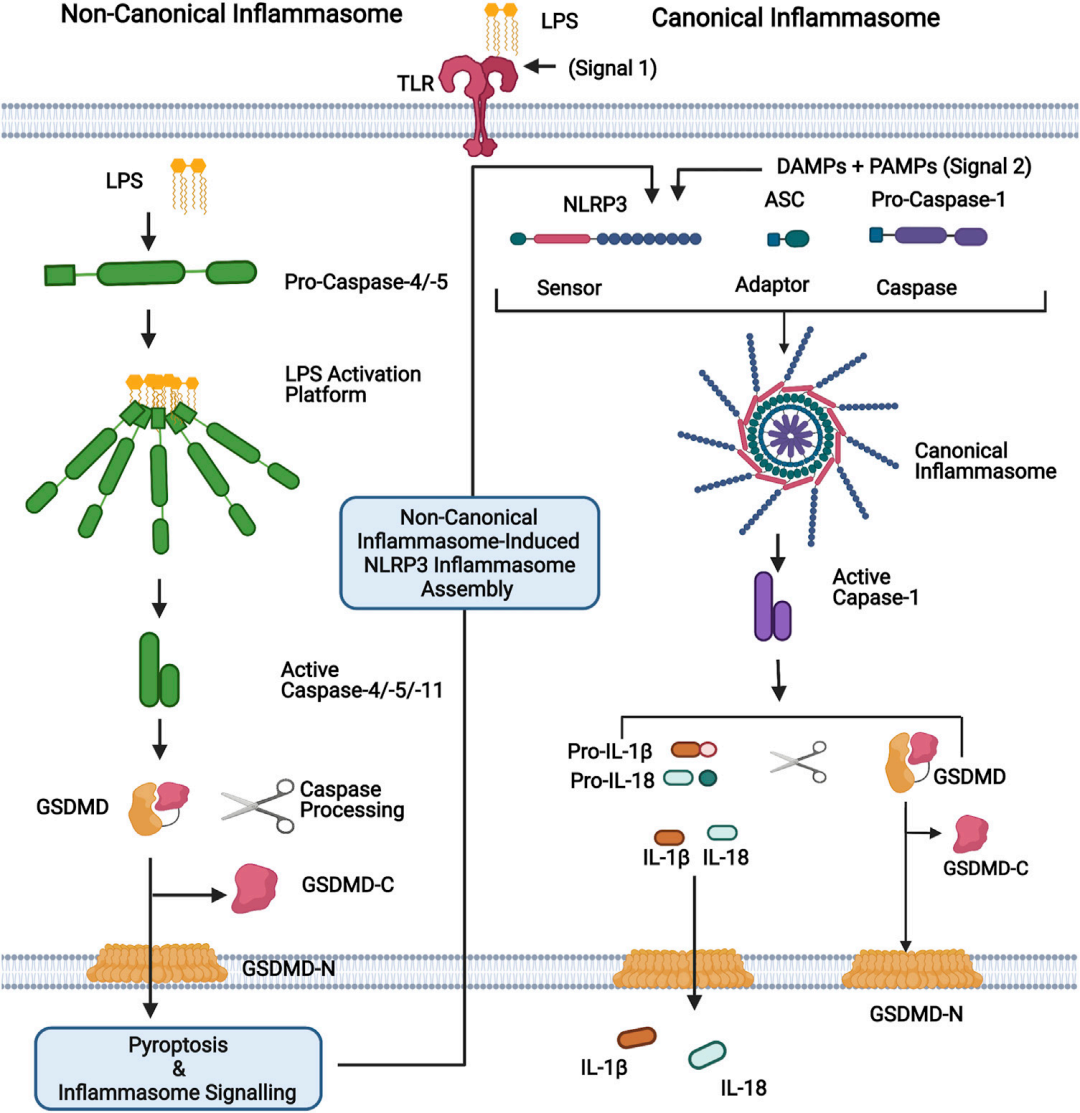
Representation of non-canonical and canonical inflammasomes. For the non-canonical inflammasome, cytosolic LPS binds directly to the N-terminal CARD domain of caspases-4, -5 (human) or -11 (mouse), leading to their activation. Active caspases-4/-5/-11 cleave their substrate, GSDMD, generating the pore forming N-terminal domain (GSDMD-N). Pore formation allows the secretion of small molecules, including potassium ions, and ultimately leads to pyroptosis. The canonical inflammasome is a multiprotein complex that forms in response to PAMPs and DAMPs. It consists of a sensor protein (eg. NLRP3 depicted here) and an adaptor (ASC) that facilitates the recruitment and activation of caspase-1. Activated caspase-1 promotes the cleavage of GSDMD and the inflammatory cytokines IL-1β and IL-18, which are subsequently released via GSDMD-N pores. The non-canonical inflammasome promotes the activation of the canonical NLRP3 inflammasome for inflammatory cytokine processing and hence activation of caspases-4,-5 and -11, which are referred to as non-canonical inflammasomes. Figure was created using BioRender.

Inflammasome sensor proteins are a class of PRRs that have been identified as playing an important role in intestinal immunity (Xu et al., 2021). Canonical inflammasomes are multiprotein complexes formed in response to PAMPs and DAMPs which consist of a sensor, an adaptor [apoptosis-associated speck-like protein containing a caspase recruitment domain (ASC)], and pro-caspase-1. Inflammasome assembly results in the processing of pro-caspase-1 into a functional heterodimer which processes the inflammatory cytokines, pro-IL-1β, and pro-IL-18 into their active forms. Active caspase-1 also promotes a lytic form of cell death termed pyroptosis through the cleavage of pore-forming protein gasdermin-D (GSDMD). GSDMD pores facilitate the release of IL-1β and IL-18, while cell lysis further promotes inflammatory signalling through the release of DAMPs (Shi et al., 2015a; Broz and Dixit, 2016; Liu et al., 2016; Van Opdenbosch and Lamkanfi, 2019) (Figure 1). Multiple sensor proteins capable of forming canonical inflammasomes have been identified including NOD-like receptor (NLR) Family Pyrin Domain Containing 1 (NLRP1), NLRP3, NLRP6, NLR Family CARD Domain Containing 4 (NLRC4), Pyrin, and absent in melanoma 2 (AIM2) (Broz and Dixit, 2016; Xu et al., 2021). Deficiency in NLRC4, NLRP6, Pyrin and AIM2 is reported to aggravate the symptoms of dextran sulphate sodium (DSS)-induced colitis, a common mouse model used to emulate acute colitis (Chen et al., 2011; Carvalho et al., 2012; Ratsimandresy et al., 2017; Sharma et al., 2018). While NLRP1 and NLRP3 deficiency has been reported to both exacerbate and protect against DSS-induced colitis (Zaki et al., 2010; Li et al., 2014; Williams et al., 2015a; Yao et al., 2017; Tye et al., 2018). IL-1β is increased in the serum of IBD patients and is associated with increased disease activity (Ligumsky et al., 1990; Kuboyama, 1998). In juxtaposition, IL-18 plays an important role in maintaining GI barrier integrity due to its epithelial repair function (Zaki et al., 2010; Chen et al., 2011; Huber et al., 2012; Oficjalska et al., 2015; Ratsimandresy et al., 2017).

In addition to the canonical inflammasome, a second class of inflammasome has been characterised, termed the non-canonical inflammasome. So called due to its secondary induction of canonical NLRP3 inflammasome assembly. Contrary to the canonical inflammasome, the non-canonical inflammasome directly senses intracellular LPS instead of associating with an upstream sensor and is composed of caspase-4 or -5 in humans and caspase-11 in mice. Recognition of cytosolic LPS by non-canonical caspases results in inflammasome assembly, caspase-4/5/11 auto-processing and the initiation of inflammatory signalling (Shi et al., 2014; Broz and Dixit, 2016) (Figure 1). Analogous to caspase-1, caspases-4, -5, and -11 promote pyroptosis through the cleavage of GSDMD, however, they are unable to directly process IL-1β and IL-18 (Shi et al., 2014; Shi et al., 2015b). Nevertheless, non-canonical inflammasome activation triggers the subsequent assembly of the NLRP3 inflammasome, which processes these cytokines (Kayagaki et al., 2011; Schmid-Burgk et al., 2015) (Figure 1).

Gram-negative bacteria are ubiquitous within the intestine, however, under non-pathogenic conditions the host cell cytosol rarely encounters LPS, as it is separated from the extracellular space and intracellular organelles by lipid membranes. Virulence factors of pathogenic bacteria and cytosol invasive bacteria include pore-forming toxins which disrupt the plasma membrane or secretion systems which introduce bacterial effector molecules from vacuolar compartments into the cytosol (Kobayashi et al., 2013; Casson et al., 2015; Matikainen et al., 2020). Thus, as intracellular LPS sensors, human caspases-4 and -5 (and murine caspase-11) and subsequent non-canonical inflammasome activation perform a unique role in differentiating between pathogenic and non-pathogenic bacteria within the GI mucosa (Casson et al., 2015).

Increasing rates of IBD in previously low prevalence countries has been linked with their adoption of a Westernised lifestyle and diet (Molodecky et al., 2011; Ng et al., 2017; Rizzello et al., 2019). The Western diet is roughly composed of 50% carbohydrate, 15% protein and 35% fat, and is classified as a high fat diet (HFD) (Last and Wilson, 2006; Schwingshackl and Hoffmann, 2013; Ye et al., 2020). Multiple studies have identified the HFD as one of the most common reasons for being overweight or obese and recent evidence suggests that a HFD is associated with gastrointestinal motility disorders, such as IBD (Schwingshackl and Hoffmann, 2013; Ye et al., 2020). A recent study investigating the mechanism which links the Western diet with intestinal dysmotility, found that increased pyroptosis occurred in colonic myenteric neurons from overweight and obese humans (Ye et al., 2020). Mice fed a Western diet also had increased neuronal pyroptosis and intestinal dysmotility, which was rescued in caspase-11 deficient mice, marking caspase-11 as a key driver of this process (Ye et al., 2020). Using *in vitro* enteric neuronal cultures and fluorescent labelling techniques, the authors showed that palmitate, a fatty acid that makes up a high proportion of the Western diet, forms lipid rafts and associates with LPS. The lipid rafts faciltate LPS entry into the cytosol and activate the non-canonical inflammasome, leading to neuronal pyroptosis. This mechanism was confirmed by experiments which showed that disruption of lipid rafts prevented LPS entry, inflammatory caspase activation and pyroptosis (Ye et al., 2020). This study therefore provides the first mechanistic insight into how an enviromental factor such as diet can affect non-canonical caspase activation in the gut, and highlights the potential for targetting inflammatory caspase activity to limit the debilitating effects of IBD.

Caspase-4 and -5 protein and RNA expression is elevated in IBD patients in active disease versus remission, suggesting that caspase-4 and -5 expression is induced directly or indirectly by intestinal inflammation (Williams et al., 2015b; Flood et al., 2015). In addition to disease activity, stromal expression of caspase-4 and -5 also positively correlates with inflammation scores, linking expression to severity of inflammation (Flood et al., 2015). Caspase-11 expression is also increased during DSS-induced colitis models, with expression being protective in this acute model of IBD (Demon et al., 2014; Williams et al., 2015b; Oficjalska et al., 2015). Caspase-11 mediated protection is associated with increased levels of IL-22 and IL-18 which are required to promote intestinal epithelial proliferation and repair (Oficjalska et al., 2015). As caspase-4 and -5 are the human orthologs of murine caspase-11 (Agnew et al., 2021), murine models have facilitated the majority of research carried out to date concerning the non-canonical inflammasome in GI inflammation and IBD. However, distinct differences exist among human and murine non-canonical caspases. This review will therefore attempt to highlight these differences, focussing on human non-canonical caspase biology and speculate how distinct aspects of this biology may be significant to IBD pathogenesis. Specifically, caspase-4 and -5 expression, and mechanisms of their function, activation and regulation that share overlapping features with IBD pathogenesis will be discussed, to potentially highlight connections between human non-canonical caspase function and IBD.

## Caspase-4 and -5 Expression

Given the crucial role of caspase-4 and -5 in mucosal immunity, their expression is stringently regulated to prevent inappropriate or overt inflammation. In human monocytes, the caspase-4 gene (*CASP4*) is constitutively expressed, and a recent genome-wide screen identified that interferon (IFN) regulatory factor 2 (IRF2) transcriptionally regulates basal *CASP4* expression. Furthermore, IFN-γ priming can compensate for IRF2 deficiency, inducing *CASP4* via IRF1, revealing a redundancy between IRF1 and IRF2 which likely exists to ensure that caspase-4 is robustly expressed following macrophage activation (Kajiwara et al., 2014; Benaoudia et al., 2019). Caspase-4 has also been reported to be constitutively expressed in macrophages and neutrophils and is also expressed in various epithelial and endothelial cells (Martin and Panja, 2002; Bian et al., 2009; Binet et al., 2010; Sansanwal et al., 2010; Sollberger et al., 2012; He et al., 2019; Cheng et al., 2021; Meng et al., 2021). Our group has shown that, in the context of intestinal epithelial cells (IECs), caspase-4 expression is restricted to neoplastic tissue, representing a novel IEC-specific biomarker for CRC diagnosis (Flood et al., 2015).

In contrast to *CASP4*, expression of the caspase-5 gene (*CASP5*) is low in normal tissues and is undetectable in resting macrophages (Lin et al., 2000; Kajiwara et al., 2014). Bacterial LPS strongly induces expression of *CASP5* mRNA and protein in monocytic cells (Lin et al., 2000). Although studies investigating *CASP5* induction are limited, another study has shown *CASP5* to be induced by IFN-γ and NFκB in psoriatic skin (Salskov-Iversen et al., 2011). Taken together*, CASP5* expression is analogous to *Casp11*, which also requires induction (Agnew et al., 2021). The divergent regulation of *CASP4* and *CASP5* supports the view that these genes are non-redundant and puts into question the validity of extrapolating results from murine colitis models to develop human therapeutics, particularly as the constitutive expression of caspase-4 cannot be replicated in this model.

## Alternative Expression of Caspases-4 and -5 During Disease?

**FIGURE 2:**
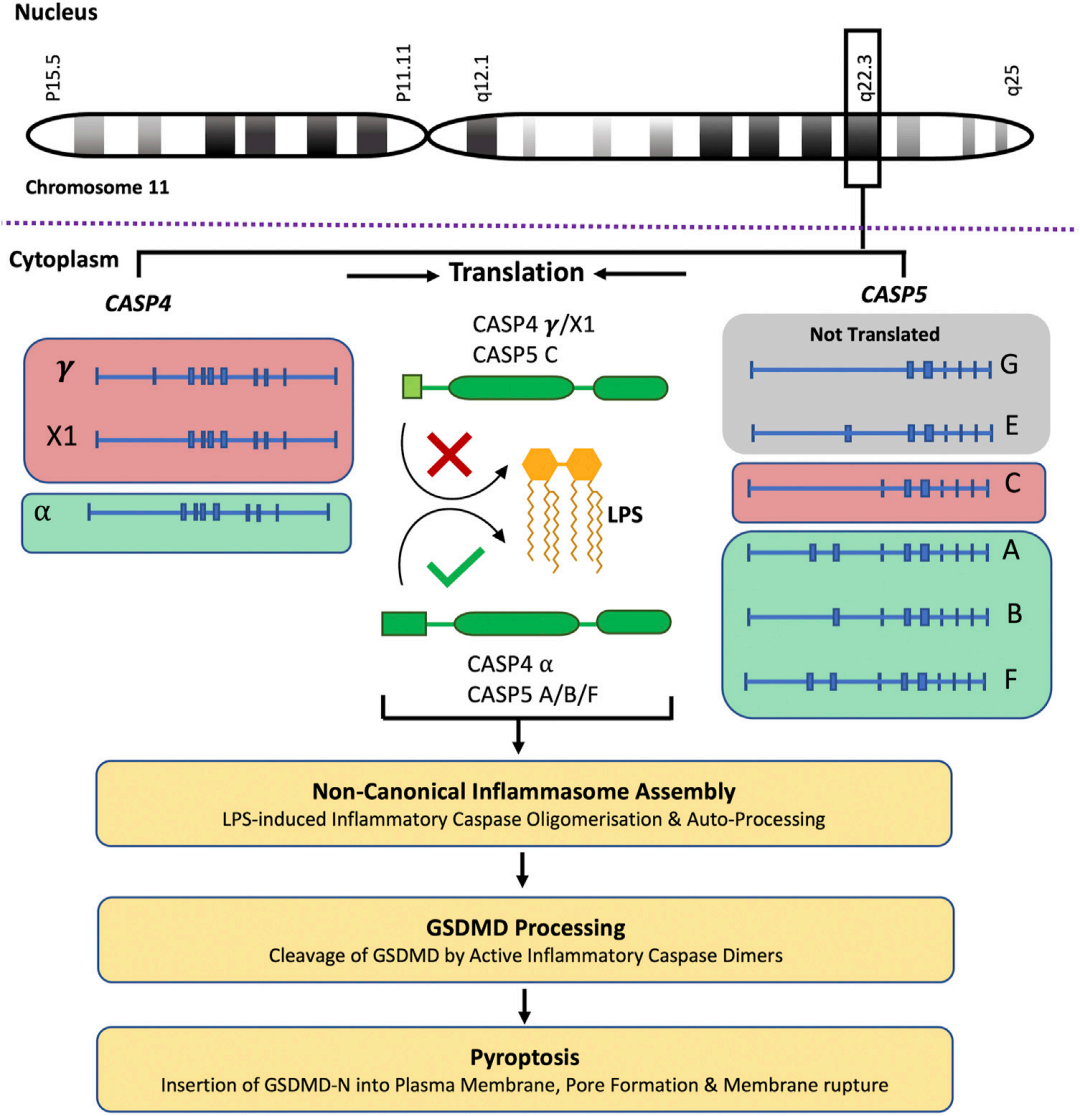
Caspase-4 and -5 variants and predicted functions. The *CASP4* and *CASP5* genes are located next to each other on chromosome 11, q22.3. NCBI RefSeq database lists three *CASP4* mRNA (α, γ and X1) which are translated into their cognate protein variants, and six *CASP5* mRNA (A, B, C, E, F and G) transcripts. Although *CASP5* codes for 6 mRNA transcripts, the G and E transcripts are not translated. The *CASP4* (γ and X1) variants and the *CASP5* (C) variant are shortened at their N-terminal regions, resulting in the loss of residues identified as being crucial for LPS binding and CARD oligomerisation. These changes in the CARD primary structure could significantly alter or prevent *CASP4* (γ and X1) variants and *CASP5* (C) variant activation in response to intracellular LPS, limiting their subsequent ability to process GSDMD and induce pyroptosis. This analysis predicts that only one *CASP4* isoform is capable of GSDMD cleavage, while *CASP5* expresses multiple potentially active isoforms.

*CASP4* (geneID:837) spans from 104,942,866–104,968,596 on chromosome 11, while *CASP5* (geneID:388) is located next to the caspase-4 gene on chromosome 11 and spans from 104,994,243–105, 023,168. Both genes are composed of ten exons which are alternatively spliced to code for different caspase-4 and -5 mRNA and protein variants (www.ncbi.nlm.nih.gov). Although caspase-4 and -5 are referred to as single proteins, like most genes *CASP4* and *CASP5* code for several variants. The proteins referred to in the above sections refer to the canonical isoforms of caspase-4 (α) and -5 (A) (Morales et al., 2022). The National Centre for Biotechnology Information (NCBI) Reference Sequence (RefSeq) database lists three *CASP*4 mRNA (α, γ and X1) and six *CASP*5 mRNA (A, B, C, E, F and G) transcripts (www.ncbi.nlm.nih.gov/protein) (Table 1). All three CASP-4 mRNA sequences code for their cognate protein, however caspase-4 variants γ and X1 are shortened at their N-terminal regions, resulting in the loss of residues identified as being crucial for LPS binding and CARD oligomerisation (discussed further in later section). These changes in the CARD primary structure could significantly alter or prevent caspase-4 γ and X1 variant activation in response to intracellular LPS, which could significantly influence gut homeostasis. Only four of the six *CASP5* mRNA transcripts are thought to be actively translated into protein *in vivo* (A, B, C and F). Variant E and G are both susceptible to nonsense-mediated mRNA decay (NMD) due to a lack of start codon (G) and a frame shift resulting in premature stop codon (E). The C variant is the only caspase-5 protein variant missing residues identified as vital for its enzymatic activity. The data presented by NCBI implies that only one caspase-4 isoform is capable of GSDMD cleavage, while *CASP5* expresses multiple active isoforms (Figure 2). Of course, other primary, secondary, and tertiary structures that impact the enzymatic activity or function of caspase-4 and -5 variants may exist. Modelling of *CASP4* variant protein structures could therefore be illuminating.

**TABLE 1:** NCBI accession numbers for Caspase-4 and -5 variants.

	Variant	mRNA accession	Protein accession
Caspase-4	α	NM_001,225.4	NP_001,216.1
	γ	NM_033,306.3	NP_150,649.1
	X1	XM_011,543,019.2	XP_011,541,321.1
Caspase-5			
	A	NM_004,347.5	NP_004,338.3
	B	NM_001,136,109.3	NP_001,129,581.1
	C	NM_001,136,110.3	NP_001,129,582.1
	E	NR_024,239.3	-
	F	NM_001,136,112.3	NP_001,129,584.1
	G	NR_036,562.3	-

Differential gene expression functions as a mode of gene regulation where different variants are expressed in a tissue-dependent manner and the gene-mediated cell responses are not driven by a single protein but by the sum of the activities of the expressed isoforms in each tissue. All *CASP5* protein coding transcripts and the *CASP4* transcripts γ and X1 (but not the α transcript) contain an upstream open reading frame (uORF). uORFs have important implications for gene expression and usually repress protein translation through ribosome stalling and blockage of the preinitiation complex (PIC) (Schuster and Hsieh, 2019). However, in stressed cells uORFs can mediate relative increases in stress-related protein expression. Activation of the integrated stress response (ISR) culminates in the phosphorylation of the transcriptional initiator eukaryotic initiation factor 2α (eIF2α) leading to global arrest of translation (Ravindran et al., 2016). In this context, PICs are likely to scan past uORFs in an mRNA transcript, leading to increased translation at the previously repressed primary start codon and resulting in a relative increase of a previously repressed protein variant (Schuster and Hsieh, 2019). The presence of uORFs in the caspase-4 and -5 transcripts could therefore influence their protein expression during stressful conditions, such as IBD and colorectal cancer. A study by Okazaki *et al.* found that inhibition of eIF2α dephosphorylation ameliorated murine experimental colitis, implying that activation of ISR can limit intestinal inflammation in this context (Okazaki et al., 2014). Ravindran *et al.* also found that deletion of the amino acid sensor and ISR inducer, general controlled non-repressed kinase (GCN2), in murine IECs and immune cells led to enhanced intestinal inflammation and DSS-induced colitis symptoms. The enhanced inflammation was linked to increased ROS production and canonical inflammasome activation (Ravindran et al., 2016). However, the group did not determine whether caspase-11 activity or non-canonical inflammasome activation was affected (Ravindran et al., 2016). This analysis suggests that the expression of alternative caspase-4 and -5 variants may be induced during diseases such as IBD. However, the cell specific expression patterns of caspases-4 and -5 and their variants remain widely unexamined. Given that cell-type-specific differences in the inducible expression of caspase-4 and -5 have been observed, it is possible that the same can be extended to expression of different variants especially during ISR (Lin et al., 2000; Salskov-Iversen et al., 2011; Kajiwara et al., 2014; Yang et al., 2015; Benaoudia et al., 2019). Elucidating the expression patterns of caspase-4 and -5 variants as well as the factors influencing these may help clarify how caspase-4 and -5 collaborate during IBD and other disease states in which their expression is altered (Williams et al., 2015b; Flood et al., 2015).

## LPS Directly Activates Caspases-4 and -5

LPS functions as a major PAMP which alerts the immune system to the presence of Gram-negative bacterial pathogens. As cytosolic LPS sensors, caspase-4 and -5 are essential in host defence against Gram-negative bacteria (Knodler et al., 2014; Casson et al., 2015). Pyroptosis as well as the release of cytokines and DAMPs facilitate bacterial clearance. Activation of caspases-4, -5, and -11 occurs when cytoplasmic LPS binds their CARD domains inducing their oligomerisation and activation. This represents a new paradigm in innate immunity, as non-canonical inflammasome caspases function both as intracellular PRRs and immune effectors (Shi et al., 2014). Activation of the canonical NLRP3 inflammasome in macrophages and dendritic cells (DCs) by contrast requires two distinct signals; 1) a priming stimulus that activates the NFκB pathway such as LPS which increases transcription of NLRP3 and Pro-IL-1β as well as 2) a second signal which induces the assembly of the inflammasome complex (Bauernfeind et al., 2009; Laudisi et al., 2013). For LPS to activate human non-canonical inflammasome signalling it must be present in or transported into the cytosol. Pathogenic virulence factors as well as cytosol invasive bacteria transport LPS into the cytosol. In 2015, Casson *et al.* confirmed that like caspase-11, caspase-4 mediates pyroptosis and IL-1β secretion in human macrophages infected with intracellular Gram-negative bacteria expressing either type III or type IV secretion systems including *Legionella pneumophila*, *Yersinia pseudotuberculosis*, and *Salmonella enterica* serovar Typhimurium (*S.*Typhimurium) (Casson et al., 2015). Although both caspase-4 and -5 bind directly to LPS *in vitro*, caspase-5 was not found to play a role in the detection of these pathogens, providing yet more evidence of distinct situational-specific functions for caspase-4 and -5 in human macrophages (Casson et al., 2015).

**FIGURE 3:**
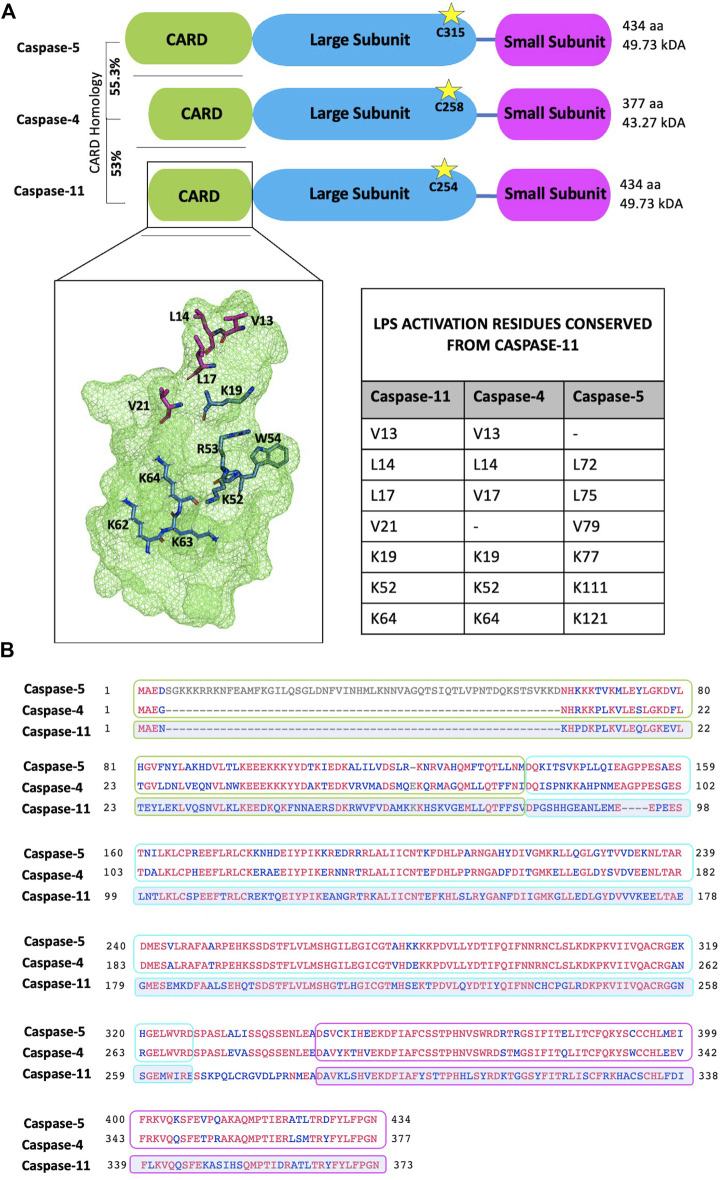
Protein domain and sequence alignments of the non-canonical caspases. **(A)** Schematic representation of caspases-4, -5 and -11. Cleavage is dependent on the conserved catalytic cysteine residue (indicated by stars). The caspase-5 N-terminal pro-domain contains an additional 58 amino acids and shares 55.3% homology with the caspase-4 pro-domain. Similarly, the caspase-4 pro-domain shares 53% homology with the caspase-11 pro-domain. Zymogen cleavage separates the CARD domain (green box), the large subunit (blue box) and the small subunit (pink box). The small and large subunits form the enzymatically active heterodimer. Crystal structure of the caspase-11 CARD highlights residues important for activation: LPS binding (blue) and oligomerisation (magenta). Residues 62-64 are modelled as they are not represented in the crystal structure. Activation residues from caspase-11 that are conserved in caspases-4 and -5 are listed in the table inset. Residues visualised and modelled using PyMol. **(B)** Sequence alignment of canonical caspase-4, -5 and -11 sequences. Conserved residues are indicated in red; grey residues are unique to caspase-5; and blue indicates non-conserved residues. In this alignment caspase-4 and -5 protein sequences are directly compared, while caspase-11 conservation is measured against caspase-4 and -5. Therefore, only residues maintained in both caspase-4 and -5 will appear red in the caspase-11 alignment. Sequence alignment was performed using the NCBI multiple alignment tool, COBALT.

As mentioned in the introductory section, caspases undergo auto-proteolysis and form obligate heterodimers for substrate recognition and processing (Van Opdenbosch and Lamkanfi, 2019). Inflammasome complexes sense activation signals and induce caspase-1 processing. Caspase-8 has also been implicated in inflammasome activation and IL-18/IL-1β production, reviewed in detail elsewhere (Monie and Bryant, 2015; Rathinam and Chan, 2018). In contrast, non-canonical inflammatory caspases-4, -5 and -11 directly interact with intracellular LPS through their CARD domains to trigger CARD oligomerisation and proximity induced auto-proteolysis, which separates the N-terminal CARD domain from the C-terminal catalytic domain (Van Opdenbosch and Lamkanfi, 2019). The catalytic domain is then further processed into large and small subunits which form the enzymatically active caspase heterodimer (Van Opdenbosch and Lamkanfi, 2019). In a recent study Liu *et al* solved the crystal structure of the caspase-11 CARD domain. Analysis of this revealed an extensive hydrophobic interface formed by V13, L14, L17 and V21 (the H1-2 alpha helix) which mediated CARD domain oligomerisation (Figure 3A). Mutation of this helix abolishes both CARD oligomerisation and the ability of caspase-11 to induce pyroptosis (Liu et al., 2020). The H1-2 residues are mostly conserved apart from V21 - > F21 in caspase-4 and V13 - > M71 in caspase-5. Another study identified caspase-11 mutations (K19E; K52E/R53E/W54A (KRW); and K62E/K63E/K64E (KKK)) which severely disrupted LPS binding. The Lysine residues (apart from K62) are conserved among caspase-4, -5 and -11, supporting the importance of these residues and a charge interaction in LPS-caspase binding (Shi et al., 2014) (Figures 3A,B).

**FIGURE 4:**
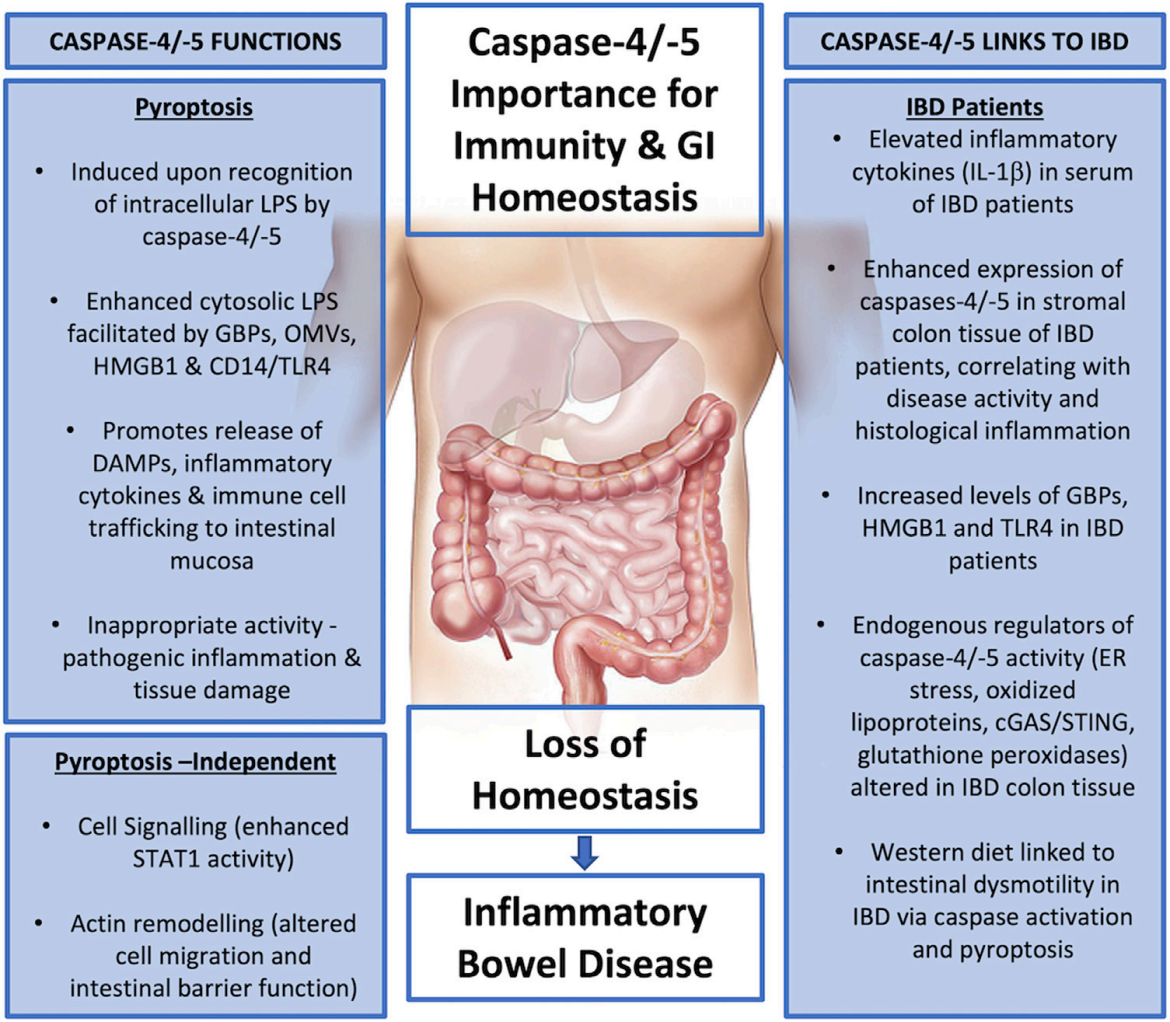
Summary diagram - Caspase-4 and -5 functions and links to IBD pathogenesis.

Caspase-4 exhibits a broader reactivity to LPS than caspase-11, triggering pyroptosis in response to both hexa- and tetra-acylated lipid A moieties of LPS, while hexa-acylated lipid A is required for caspase-11-mediated pyroptosis (Lagrange et al., 2018). This discrepancy is highly likely to be influenced by differences within the CARD domains of caspase-4 and -11, which only share 53% sequence identity (Figure 3B). Yet, the mechanisms underpinning LPS-caspase binding were recently revealed to be more complex. *Chu et al.* found that not only positive CARD residues but also positive residues within the large subunit of caspase-11, between amino acid 220 to 294 were necessary for LPS binding (Chu et al., 2018). As humans express two enzymes (caspase-4 and -5) instead of one (caspase-11) the prevailing dogma has been that caspase-4 and -5 are redundant. However, increasing evidence suggests independent roles for caspases-4 and -5. For instance, considerable heterogeneity is observed at the N-terminal CARD domain of caspase-4 and -5 which share only 55.3% identity (Figure 3B). Considering this, and the differences in LPS sensitivity seen between caspase-4 and -11, it is conceivable that the extended N-terminal domain of caspase-5, containing the CARD, may affect how LPS binds and therefore activity of caspase-5 (Figure 4).

## Mechanisms Enhancing LPS-Mediated Activation

Additional modes of LPS-mediated cytosolic activation of caspase-4 and -5 have also been characterised, as discussed below:

### Enhanced Caspase-4 Activation Facilitated by GBPs During IBD?

Guanylate binding proteins (GBPs) are IFN-inducible GTPases reported to function as LPS sensors that act upstream of caspase-4 in cytosolic LPS sensing in human epithelial and immune cells (Santos et al., 2018; Wandel et al., 2020). Specifically, GBP1 binds LPS with high affinity via electrostatic interactions between negatively charged groups on LPS and a positive K61, K62 and K63 patch in the GTPase domain of GBP1. GBP1 assembles a GBP coat by recruiting GBPs2-4. The GBP coat recruits caspase-4 to cytosol-invasive bacteria or LPS micelles, promoting caspase activation. In addition to achieving sufficiently high concentrations of caspase-4 at the bacterial surface to facilitate its activation, GBPs also seem to make lipid A more accessible by disrupting the bacterial cell envelope (Kutsch et al., 2020; Santos et al., 2020; Wandel et al., 2020). In contrast to this human-specific mechanism, previous murine models had reported that GBPs promote inflammasome activation through vacuolar disruption, which facilitates bacterial escape and direct bacteriolysis (Meunier et al., 2014; Santos et al., 2018). Additional recruitment of pore-forming immunity related GTPases (IRGs) such as IRGb10 by murine GBPs may explain vacuole disruption and bacteriolysis seen in these models (Man et al., 2016). In addition to its roles in immune regulatory function and epithelial barrier defects, IFN-γ has been shown to contribute to vascular leakage, all of which sustain and perpetuate inflammation in the context of IBD (Haep et al., 2015; Langer et al., 2019). Patients with active CD or UC show increased expression of GBP-1 mRNA and protein in colon tissue when compared to healthy controls, and GBP-1 has been proposed as a marker for IFN-γ activity in IBD patients (Haep et al., 2015). Similarly, murine colitis models result in higher GBP-1 expression in GI tissue (Haep et al., 2015). It is therefore plausible to suggest that GBP-1 facilitates caspase-4 activation in the stromal tissue of IBD patients during active disease (Flood et al., 2015; Haep et al., 2015).

### OMVs–Recruitment of Caspase-5 and Links to Barrier Dysfunction?

Outer membrane vesicles (OMVs) are LPS containing membrane bound structures precipitated from Gram-negative bacteria during bacterial growth and/or stress (Beveridge, 1999). OMVs are internalised via endocytosis and released from the endocytic compartment into the cytosol. A study examining non-canonical inflammasome activation in response to OMVs from *Pseudomonas aeruginosa* in THP-1 cells found that caspase-5 but not caspase-4 is activated (Bitto et al., 2018). In the same cells, caspase-4 was activated in response to free *P. aeruginosa* LPS transfection and live bacteria (Bitto et al., 2018). This again suggests that caspase-4 and -5 efficiently co-ordinate host defence using differential LPS recognition capabilities, dependent on the physical form of LPS and possibly its mechanism of intracellular entry. However, in the context of IBD the differential contributions of caspase-4 and -5 to inflammatory and other pathological events are not clear. Recently, Wang *et al.* proposed that caspase-5 may contribute to intestinal barrier dysfunction, rather than pyroptosis, during IBD (Wang et al., 2021). Sorting nexin 10 (SNX10) was shown to facilitate cytosolic release of LPS from OMVs by recruiting caspase-5 to the endosomal membrane. Caspase-5 is reported to facilitate the down regulation of E-cadherin expression in a process dependent on Lyn phosphorylation and activation of Snail/Slug transcription factors (Wang et al., 2021). Furthermore, knockdown or inhibition of SNX10 rescued OMV-induced barrier dysfunction and DSS-induced colitis symptoms in mice (Wang et al., 2021). This finding provides further evidence for the complex and varied roles of non-canonical caspases at mucosal sites and the necessity for further research into basic caspase biology and their involvement in IBD pathogenesis.

### HMGB1 Release–Amplification of Caspase Activity During IBD?

HMGB1 is a nuclear protein that plays an important role in cellular processes such as transcription, replication, and DNA repair. When released from cells it functions as an alarmin or DAMP (Deng et al., 2019). Circulating HMGB1 is increased during endotoxemia/sepsis and antibody-mediated disruption of HMGB1-LPS binding confers resistance to sepsis (Yang et al., 2004; Sundén-Cullberg et al., 2005; Youn et al., 2008). The human and murine non-canonical inflammasome has been identified as a major contributor to sepsis (Kajiwara et al., 2014; Deng et al., 2018). In murine endotoxemia and bacterial sepsis models, HMGB1 binds LPS which is subsequently recognised and internalised by receptor for advanced glycation end products (RAGE)-mediated endocytosis. HMGB1-mediated permeabilization of lysosomal membranes facilitates LPS release into the cytosol where it activates caspase-11 (Youn et al., 2011; Deng et al., 2018). Systemic HMGB1 is elevated in human septic shock patients and correlates with a worse prognosis (Hatada et al., 2005; Sundén-Cullberg et al., 2005). Therefore, it is likely that similar mechanisms are involved in cytosolic LPS delivery and caspase-4 and -5 activation in human sepsis. Pathogenesis of both sepsis and IBD involves the loss of immune homeostasis, however, sepsis represents an acute systemic response whereas IBD is a more localised chronic condition. Serum, tissue, and faecal levels of HMGB1 are higher in patients with IBD as well as in mice subjected to DSS-/Trinitrobenzenesulfonic acid (TNBS)-induced colitis (Maeda et al., 2007; Davé et al., 2009; Vitali et al., 2011). HMGB1 neutralising antibodies or antagonists improve the clinical symptoms of murine colitis (Yang et al., 2006; Maeda et al., 2007; Davé et al., 2009; Ju et al., 2014). HMGB1 secretion is dependent on plasma membrane rupture either via necrosis, necroptosis or pyroptosis (Kayagaki et al., 2021). Given that expression of caspase-4 and -5 is also increased during IBD it is worth questioning whether a portion of HMGB1 secretion can be attributed to non-canonical inflammasome signalling and that HMGB1-LPS internalisation may contribute to the sustained inflammation of the mucosa *via* activation of caspase-4 or -5, as is the case in sepsis (Williams et al., 2015b; Flood et al., 2015; Kayagaki et al., 2021).

### CD14/TLR4-Mediated LPS Internalisation–Enhanced During IBD

LPS sensitivity is reported to be several orders of magnitude higher in humans than in mice (Fink, 2014). As discussed previously one facet of this discrepancy can be attributed to broader sensitivity of caspase-4 to under acylated LPS (Warren et al., 2010; Lagrange et al., 2018). Human monocytes are believed to be important mediators of human sepsis, which occurs when inflammatory responses to infection are so robust that they induce physiologic alterations in the host (Fingerle et al., 1993; Stearns-Kurosawa et al., 2011). It has been proposed that monocyte pyroptosis mediates early inflammatory events which drive sepsis (Eisinger et al., 2022). Rapid monocyte recruitment into peripheral tissues during microbial infection allows for the replenishment of local macrophage and DC populations (Serbina et al., 2008). High expression of CD14, the co-receptor for the extracellular LPS receptor, TLR4, is a defining characteristic of monocytes. Upon differentiation into macrophage or DC populations, CD14 expression reduces drastically (Gordon and Taylor, 2005; Viganò et al., 2015). Taking this into account, Viganò *et al.* hypothesised that CD14/TLR4-mediated LPS internalisation may play an important role in noncanonical inflammasome activation in monocytes (Viganò et al., 2015). Interestingly, they found that caspase-5 but not caspase-4 was processed in response to CD14/TLR4 internalised LPS. This pathway required Syk activity and Ca^2+^ flux, and led to IL-1 α/β release, indicative of non-canonical inflammasome-mediated pyroptosis (Viganò et al., 2015). A meta-analysis investigating the affect of the CD14 C260T polymorphism, which is linked to increased CD14 expression, found a positive association between C260T and UC, with frequency significantly varying between caucasians and asians (48.47% vs. 65.98%) (Wang et al., 2012). A murine study reported CD14 to play a protective role in DSS-induced coilitis by enhancing intestinal barrier function during inflammation, however, in an IL-10 defincient model of IBD, CD14 expression had no effect on disease outcome, implying that CD14 may only be protective in acute settings (Buchheister et al., 2017). Although CD14 expression is reduced in tisssue resident cells, a study examining mucosal dendritic cells from 76 IBD patients and 76 healthy controls found expression of TLR4 and LPS uptake was increased in IBD patients verus healthy controls, as well as in IBD patients in remission verus active disease, suggesting that enhanced LPS uptake occurs in patients with IBD (Baumgart et al., 2009). Taken together, data suggest that endocytosis of extracellular LPS via TLR4/CD14 is increased in patients with IBD and may contribute to severe inflammation by causing caspase-5 mediated pyrotosis in monocyte and monocyte-derived populations.

## LPS-independent Activators of Caspases-4 and -5

Despite the importance of caspase-4 and -5 activation in pathogen clearance, their inflammatory signalling is implicated in driving numerous non-infectious inflammatory pathologies (Salskov-Iversen et al., 2011; Flood et al., 2015; Kajiwara et al., 2016; Kenealy et al., 2019; Zaslona et al., 2020a; Zasłona et al., 2020b). The exact circumstances leading to inappropriate activation of inflammatory caspases during sterile inflammation is unclear and suggests the existence of endogenous ligands. The nature of these ligands remains elusive. Below we will discuss the current potential endogenous activators of non-canonical inflammasome signalling and their possible relevance to IBD.

### Heme

Heme is a DAMP released from red blood cells, whose excessive release due to hemolysis is a key feature of several disease states, including sepsis, malaria, and sickle cell disease. Higher levels of free heme have been implicated in inflammatory activation of macrophages, monocytes, and endothelial cells (Larsen et al., 2010; Belcher et al., 2014; Vinchi et al., 2016; Bolívar et al., 2021; Vasquez et al., 2021). Bolívar *et al.* proposed that this inflammatory signalling is in part mediated by the non-canonical inflammasome (Bolívar et al., 2021). Of note, heme activated caspase-4 and -5 displayed unique and non-synonymous functions. Heme-induced pyroptosis was mediated mainly through caspase-4 activation, while heme-induced IL-1β release and caspase-1 activation was mediated by both caspase-4 and -5 (Bolívar et al., 2021). Heme oxygenase 1 (HO-1) is the enzyme responsible for the breakdown of heme into eqaul parts iron, carbon monoxide and biliverdin. A study investigating the role of HO-1 in macrophages during *S. typhimurium* infection found that HO-1 knockdown significantly enhanced bacterial clearance, which was caused by reduced bacterial uptake (Mitterstiller et al., 2016)*.* Moreover, heme levels correlate with worse prognosis in sepsis patients and HO-1 has been shown to be protective in a murine sepsis model (Larsen et al., 2010). Given the previously discussed importance of the non-canonical inflammasome in sepsis and controling intracellular Gram-negative bacterial pathogens*,* these findings suggest that HO-1 limits the activation of non-canonical inflammasome signalling by modulating heme levels (Kajiwara et al., 2014; Casson et al., 2015; Deng et al., 2018).

### oxPAPC

Oxidised 1-palmitoyl-2-arachidonoylsn-glycero-3-phosphoryl-choline (oxPAPC), a bioactive component of oxidised low-density lipoproteins, is a modulator of the inflammatory response (Karki and Birukov, 2018). oxPAPC is elevated in GI tissue and plasma of patients with CD (Kruidenier et al., 2003; Alzoghaibi et al., 2007; Egea et al., 2017). Consistent with these human findings, oxPAPC is eleveated in the cyclooxygenase-2 and myeloid knockout murine models of Crohn’s-like inflammation and was hypothesised to amplify intestinal inflammation (Meriwether et al., 2019). The specific role of oxPAPC seems to be concentration and context dependent, as dual pro- and anti-inflammatory effects have been described. OxPAPC acts as a TLR4 agonist, inducing the expression of IL-6, IL-8 and CCL2 in monocytes (Walton et al., 2003; Imai et al., 2008). High concentrations of oxPAPC mediate caspase-11 oligomerization and activation in murine bone marrow-derived dendritic cells (Zanoni et al., 2016). Thus, it is possible that high levels of oxidised phospholipids in IBD patients may contribute to the activation of caspase-4 or -5.

### ER Stress

The cellular state in which demand for endoplasmic reticulum (ER) function exceeds its capacity is referred to as ER stress. The ER oversees the folding, maturation, and storage of membrane and secreted proteins. Furthermore, it acts as a storage organelle for Ca^2+^ and therefore is an important player in sensing perturbations in cellular homeostasis. During inflammatory diseases such as IBD, cells can be pushed into a state of ER stress as they are unable to keep up with protein demand induced by inflammatory signalling (Eugene et al., 2020). Early studies suggested that ER stress was an activator of caspase-4, which could trigger ER stress-induced apoptosis (Hitomi et al., 2004; Katayama et al., 2004; Jiang et al., 2007). Use of caspase-4 peptide inhibitors, siRNA depletion, or expression of a catalytically inactive caspase-4 led to significantly decreased ER-stress induced apoptosis in various cells/cell lines (López-Antón et al., 2006; Chen et al., 2008; Oda et al., 2008; Pataer et al., 2008). As many of these studies appeared before caspase-4-mediated pyroptosis had been characterised and as pyroptosis shares morphological characteristics with apoptosis, such as TUNEL staining and chromatin condensation, it is possible that pyroptotic and not apoptotic death is occurring. However, later studies showed evidence of caspase-4-mediated procaspase-9 processing and caspase-3 mediated apoptotic execution (Hengartner, 2000; Yamamuro et al., 2011). Other studies suggest that inflammasome generated GSDMD-N fragments can permeabilise mitochondria, further linking the inflammasome with the induction of apoptosis through the release of cytochrome-c and activation of caspase-3 (Rogers et al., 2019). Evidence also exists that apoptotic caspases participate in pyroptotic cell death through their actions on members of the gasdermin family, thus, it is possible that caspases can fulfil interchangeable roles within a cell, dependent on context and stimulus (Wang et al., 2017; Zheng et al., 2020). Could induction of apoptosis, rather than pyroptosis, by caspase-4 in response to ER stress represent a cellular mechanisms to limit further inflammatory signalling and therefore ER stress in neighbouring cells? Although the role of caspase-4 in apoptosis requires further investigation, these studies suggest that ER stress may function upstream of caspase-4 as an endogenous activator during IBD.

### cGAS/STING

Caspase-4 activation and GSDMD-dependent cell death have been proposed to drive retinal pigmented epithelium (RPE) degeneration during geographic atrophy, an advanced form of age-related macular degeneration (Kerur et al., 2018). Caspase-4 activity in this model was driven by mitochondrial damage and activation of Type I IFN, via cyclic GMP-AMP synthase (cGAS) and stimulator of interferon genes (STING) (Kerur et al., 2018). While caspase-4 expression has previously been shown to be IFN-inducible, this study provides the first evidence of cGAS acting as an upstream caspase-4 activator, expanding the pathological role of caspase-4 to non-infectious inflammatory diseases (Kajiwara et al., 2014; Kerur et al., 2018; Benaoudia et al., 2019). Murine models further substantiate a role for STING signalling in inflammasome activation. Zhang *et al.* demonstrated that STING-mediated Ca^2+^ release drives GSDMD processing and pyroptosis (Zhang et al., 2020). STING-dependent signalling drove lethal coagulation in murine sepsis through the release of coagulation factor III (F3), a key initiator of blood coagulation, further widening the scope of functional outcomes of caspase activation following severe bacterial infections (Zhang et al., 2020). *TMEM173,* the gene that codes for STING, has been found to be hypomethylated in the GI epithelium of pediatric IBD patients, which could lead to overexpression and overactivity of STING in IECs from IBD patients (Howell et al., 2018). In support of this theory, IBD patients express a wide variety of IFN-regulated genes (IRGs) with higher IFN signatures associated with therapeutic failure (Coskun et al., 2013; Andreou et al., 2020; Wottawa et al., 2021). A recent paper further links cGAS and inflammsome signalling to colitis (Ma et al., 2020). Increased expression and processing of caspase-11 and GSDMD were observed during DSS-induced colitis. GSDMD deficiency aggravated the coilitis phenotype, in line with other studies investigating the outcome of inflammasome signalling in DSS-induced coilitis (Zaki et al., 2010; Chen et al., 2011; Carvalho et al., 2012; Williams et al., 2015a; Ratsimandresy et al., 2017; Yao et al., 2017; Sharma et al., 2018). The study proposes a physiological role for non-canonical inflammasome and GSDMD activation, limiting the severity of experimental DSS-colitis models by restricting cGAS-mediated inflammation in macrophages, independent of microbiota-associated changes or the production of antimicrobial peptides (Ma et al., 2020). Whether a similar physiological role may exist for non-canonical caspases and GSDMD in controlling cGAS-mediated inflammation, in the context of IBD developent in patients, has yet to be explored.

### Functional Outcomes of Caspase-4 and -5 Activation

#### Pyroptosis–The Inflammatory Cell Death Process

Although the active site of inflammatory caspases is highly conserved, inflammatory caspases can have distinct substrate specificities. As described earlier, caspase-4, -5 and -11 cleave GSDMD to induce pyroptosis but are incapable of efficiently processing pro-IL-1β and pro-IL-18, which are processed by caspase-1. Recent studies have begun to decipher the mechanisms of the distinct inflammatory caspase substrate specificities given the relative active site conservation. A study using fluorogenic peptide substrates found that caspase-4, -5 and -11 substrate specificity for GSDMD is influenced by prime side amino acids P1′- P4′, while caspase-1 substrate affinity is mostly influenced by the P1-P4 sequence (Bibo-Verdugo et al., 2020). Prime side amino acids refer to the C-terminal region of a substrate adjacent to the cleavage site, while P1-P4 refer to the N-terminal amino acids (Bibo-Verdugo et al., 2020). Unfortunately, the specific residues involved in forming prime side pockets are yet to be elucidated, and prime side residues are very poorly represented in available caspase structures.

GSDMD processing involves its cleavage after the aspartic acid residue 276 (Shi et al., 2015b). Cleavage separates the inhibitory GSDMD-C domain from the pore forming GSDMD-N domain, resulting in GSDMD-N insertion into the lipid bilayer, oligomerisation, and pore formation (Shi et al., 2015a; He et al., 2015). Pore formation ultimately results in cell swelling, membrane lysis, and pyroptosis (Shi et al., 2015b; Ruan et al., 2018). Ectopic expression of GSDMD-N is sufficient to induce pyroptosis, identifying it as the executioner of pyroptosis (Ding et al., 2016). Pores have an inner and outer diameter of 18 nm and 28 nm respectively, allowing secretion of small molecules such as IL-1β, IL-18, as well as potassium ions (Qu et al., 2011; Broz and Dixit, 2016; Ruan et al., 2018). Potassium efflux is a well characterised NLRP3 activating event, therefore, NLRP3 activation is a consequence of non-canonical caspase mediated GSDMD processing (Qu et al., 2011; Baker et al., 2015).

IL-1β and IL-18 as well as DAMPs such as HMGB1 lack signalling domains for cellular secretion. Therefore, smaller molecules including IL-1β and IL-18 are released through GSDMD pores while secretion of HMGB1 and other larger molecules is mediated through a loss in membrane integrity (Youn et al., 2008; Deng et al., 2018; Van Opdenbosch and Lamkanfi, 2019). Until recently plasma membrane lysis was considered a passive event, however, the cell-surface protein Ninjurin-1 (NINJ1) has been identified as a critical mediator of membrane lysis (Kayagaki et al., 2021). NINJ1 expression is protective during DSS-induced colitis as well as colitis associated carcinoma (CAC). This observation supports the homeostatic role of inflammasome signalling and pyroptosis within the GI tract, facilitating the recruitment of cells to the site of infection (Woo et al., 2016; Choi et al., 2020). However, uncontrolled caspase activation can result in widespread systemic inflammation as seen in endotoxemia/sepsis (Kumar, 2020). Furthermore, this process is not restricted to cells of the myeloid linage, Knodler *et al.* demonstrated that caspase-4 and -11 are activated in response to Gram-negative bacteria in intestinal epithelial cells (IECs) and that this activation was indispensable for pathogen clearance and GI homeostasis (Knodler et al., 2014).

### Pyroptosis Independent Functions

A number of pyroptosis-independent roles for non-canonical inflammasome associated caspases, particularly caspase-11, are emerging and are reviewed in more detail elsewhere (Downs et al., 2020; Agnew et al., 2021). These include cell signalling roles, including TRAF6 association with caspase-4 in human monocytes (Lakshmanan and Porter, 2007) and a role for murine caspase-11 in mediating indirect STAT1 activation in both IECs (following IL-1β- and LPS-stimulation) and BMDM (following LPS-stimulation) (Flood et al., 2019). In the context of the AOM/DSS colitis-associated carcinogenesis model, *CASP11* deficiency leads to increased IEC proliferation and angiogenesis, which are usually repressed by STAT1 activity (Bromberg et al., 1996). The non-canonical caspases have also been implicated with roles in regulating actin dynamics. Caspase-11 has been demonstrated to mediate phagolysosome fusion and clearance of pathogenic Gram-negative bacteria via remodelling of actin dynamics (Akhter et al., 2012; Caution et al., 2015; Krause et al., 2018). Caspase-11 deficiency is also linked to impaired neutrophil migration and reduced production of neutrophil extracellular traps in an experimental model of gout (Caution et al., 2019). Findings by Akhter *et al.* suggest that caspase-4 and -5 also regulate actin dynamics and are involved in phagolysosome fusion to facilitate the clearance of pathogenic bacteria (Akhter et al., 2012). In cancer epithelial cells, caspase-4 has been associated with the regulation of cell migration and the restoration of cell contacts through actin remodelling (Papoff et al., 2018). Further investigations to determine whether inflammatory caspase-4 and/or -5 are mediating any of these pyroptosis-independent effects in the context of IBD are therefore warranted.

Infiltrating neutrophils are a common feature in the mucosa of IBD patients and levels of infiltration have been shown to correlate with disease activity (Akpinar et al., 2018; Therrien et al., 2019). Despite their prominence in the IBD mucosa, the potential for targeting neutrophil-mediated inflammation as an IBD treatment has not been widely explored. A key inflammatory process driven by neutrophils is the generation of neutrophil extracellular traps (NETs). NETs are formed by the extrusion of chromatin, antimicrobial peptides and oxidative enzymes from neutrophils, representing an important defence mechanism against pathogens at the IBD mucosal interface (Drury et al., 2021). However, NETs can also potentiate inflammation, with significantly higher NET levels being observed during active IBD (Li et al., 2020; Drury et al., 2021). Interestingly, caspase-4 (and its murine ortholog, caspase-11) and Gasdermin D have been implicated in process of NET generation in both human and murine neutrophils (Chen et al., 2018), providing further evidence for caspase-4 as an important inflammatory mediator during IBD.

## Regulation of Caspase-4 and -5 Activity

The non-canonical inflammasome is an essential component of the innate immune system. However, the detrimental effects of overt non-canonical inflammasome signalling is illustrated in conditions such as septic shock (Kajiwara et al., 2014). Therefore, in order to maintain homeostasis, negative feedback must exist to regulate signalling and prevent inappropriate activation. In turn, to survive and successfully infect a host, pathogens must also find mechanisms to prevent activation of the non-canonical inflammasome. The simplest way for Gram-negative bacteria to evade detection is to modify their LPS, in particular the lipid A moiety. However, as human caspase-4 exhibits a broader reactivity to LPS than murine caspase-11, human pathogenic bacteria must utilise other mechanisms to antagonise inflammasome signalling (Lagrange et al., 2018). Some of the relevant host and bacterial caspase-4/-5 antagonists will be discussed below.

## Host-Derived Antagonists

As described earlier, oxPAPC has pro-inflammatory roles at high concentrations, however low concentrations closer to the physiological range have demonstrated opposing anti-inflammatory effects (Imai et al., 2008; Zanoni et al., 2016; Chu et al., 2018). At low concentrations, oxPAPC antagonises TLR4 by competing with LPS for binding (Imai et al., 2008). Similarly, binding of oxPAPC to caspase-4 in humans and caspase-11 in mice competes with LPS and functions as an inhibitor of the non-canonical inflammasome and pyroptosis (Chu et al., 2018). Anti-inflammatory actions have also been described for lysophosphatidylcholine, an oxidised low-density lipoprotein with similar structure to oxPAPC, supporting oxPAPC’s role as an immune regulator (Li et al., 2018). OxPAPC and its derivatives may therefore have therapeutic potential as regulators of non-canonical caspases during IBD.

Reactive oxygen species (ROS) have been shown to play a role in the regulation of caspase-11 expression as well as NLRP3 activation (Lupfer et al., 2014; Abais et al., 2015). Gluthione peroxidases are critical in regulating ROS within biological systems (Brigelius-Flohé and Flohé, 2020). Given this, *Hsu et al.* hypothosised that genes involved in modulating oxidative stress signals such as *GPX8,* which codes for gluthione peroxidase 8 (GPx8), may play a role in the non-canonical inflammasome pathway. *Gpx8* knockout in mice severely exacerbated DSS-colitis and reduced gut microbiome diversity (Hsu et al., 2020). Immunostaining of murine and human colon tissues revealed that GPx8 was primarily expressed in macrophages. Macrophages isolated from *Gpx8* ^*−/−*^ mice post-DSS treatment expressed higher levels of pro-inflammtory cytokines (IL-1β and IL-6) and adoptive transfer of *Gpx8*-deficient macrophages into mice prior to DSS treatment resulted in an exacerbated colitis phenotype indicating a protective role for GPx8 in macrophages during experimental colitis (Hsu et al., 2020). Upregulation of proinflammatory cytokines and aggravation of colitis symptoms in *Gpx8* ^*−/−*^ mice was found to be dependent on its action on caspase-11. Specifically, oxidized GPx8 is proposed to form a disulfide bond with caspase-11 (C79 of GPx8 with C116 of caspase-11). This binding prevents oligomerisation and activation of caspase-11, thus GPx8 functions as a redox sensor that inhibits non-canonical inflammasome signalling upon oxidisation by ROS. Importantly *Hsu et al.* demonstrated that GPx8 also covalently associates with the conserved residue in caspase-4 (C118), but not murine or human caspase-1, to inhibit caspase-4 activation in response to intracellular LPS (Hsu et al., 2020). The cysteine residue (C118) is also conserved in caspase-5, therefore it is likely that GPx8 can also regulate caspase-5 activity in response to oxidative damage. In the context of IBD this modulation of the non-canonical inflammasome may be of clinical importance as UC patients have lower *Gpx8* and higher *CASP4* expression ratios in the colon when compared to controls suggesting that GPx8 protects against coilitis by acting as a negative regulator of caspase-4/-11 (Hsu et al., 2020).

In agreement with the above findings, genome-wide association studies have identified antioxidant genes, including *GPX4,* as IBD susceptibility loci (Jostins et al., 2012; Hsu et al., 2020). Oxidative stress has also been demonstrated to induce IBD as well as CRC (Grisham, 1994). Glutathione peroxidase 4 (GPX4) is an antioxidant enzyme that protects against oxidative stress through the reduction of lipid peroxides within a cell. Global deletion of *Gpx4* is incompatible with life and results in early embryonic lethality, illustrating its importance (Imai and Nakagawa, 2003). Kang e*t al.* demonstrated that that GPX4 negatively regulates pyroptosis through phospholipase Cγ1 and Ca^2+^-dependent mechanisms in response to intracellular LPS. Pyroptosis in this model was mediated by both canonical and non-canonical inflammasomes, suggesting that lipid peroxidation may act as universal accelerator of inflammasome activation (Kang et al., 2018). The gluthione peroxidases therefore also represent endogeneous antagonists of inflammatory caspases with potential for therapeutic manipulation, to regulate inflammation in IBD patients.

## Pathogen-Derived Antagonists

Inflammatory caspases play a key role in pathogen defence by promoting inflammation and destroying the replicative niche of Gram-negative intracellular bacteria, therefore, microbes employ strategies to avoid caspase-4 and -5 mediated human host defence to facilitate infection. Apart from LPS modification (discussed earlier), bacteria can secrete virulence factors that inactivate caspases directly*. Citrobacter rodentium* and *Escherichia coli* express Nlef, a virulence factor which is injected into the host cell via a type III secretion system (T3SS). It directly binds to the catalytic domain of caspases-4 and -11, suppressing their function and resulting in impaired IL-18-mediated host defence (Blasche et al., 2013; Pallett et al., 2017). The virulence factor OspC3 from *Shigella flexneri* prevents heterodimerisation and the formation of a catalytic pocket in caspase-4, but not caspase-11 (Kobayashi et al., 2013). Infection of the GI tract with these pathogenic bacteria causes symptoms such as intestinal inflammation sometimes progressing to haemorrhagic coilitis, and therefore serves to exemplify the importance of non-canonical inflammasome signalling in protecting against pathogenic GI bacteria and maintaining GI homeostatisis (Hartland and Leong, 2013; Nisa et al., 2020). Further structural analysis of OspC3 and Nlef virulence facors, capable of inactivating human caspase-4 activtiy, would be informative for the design of small molecule inhibitors for the treatment of inflammatory disorders associated with elevated caspase activity, such as IBD.

## Concluding Summary

The detailed analysis of human and murine IBD studies provided in this review leaves little doubt regarding the involvement of non-canonical caspases-4, -5 and -11 in the inflammation and pathogenesis of IBD. Serum levels of the potent inflammatory cytokine, IL-1β, are elevated in IBD patients (Ligumsky et al., 1990), providing the first piece of evidence to support the involvement of inflammasome activity. Human caspases-4 and -5 are elevated in stromal tissue from UC patients, and their expression levels correlate with disease activity and inflammation scores (Flood et al., 2015). Furthermore, the Western diet, thought to be a key environmental factor linked to increased IBD incidence levels, has been recently proposed to cause non-canonical caspase activation and pyroptosis in enteric neuronal cells in the colon, leading to intestinal dysmotility (Ye et al., 2020).

The precise details of when and how the non-canonical inflammasome-associated caspases are expressed and become active during the development of IBD have yet to be defined. This is complex, as several transcripts of caspases-4 and -5 exist, many of which have upstream open reading frames and therefore may lead to altered expression under conditions of cellular stress, such as IBD-mediated inflammation. Furthermore, this review details the subtle but important differences in the regulation and expression patterns of caspases-4 and -5, which suggests that they have evolved separately to enhance innate immune recognition and clearance of diverse Gram-negative pathogens. While caspases-4, -5 and murine caspase-11 are all capable of directly binding to LPS, differences in their binding capabilities to alternative LPS forms exist and the route of LPS internalisation may also influence their activation, as demonstrated by the selective activation of caspase-5 by OMVs from *P. aeruginosa*, but caspase-4 from live, free *P. aeruginosa* infection (Bitto et al., 2018).

Several endogenous activators of inflammatory caspases have also been proposed, which are likely to be key contributing factors to chronic inflammatory diseases. These include oxPAPC, ER stress and cGAS/STING signalling–all of which are elevated in IBD patients and implicated in its pathogenesis (Kruidenier et al., 2003; Eugene et al., 2020; Wottawa et al., 2021). Once activated, how do inflammatory caspases contribute to IBD-mediated inflammation? The best characterised outcome of caspase-4/-5/-11 activation is GSDMD cleavage, resulting in the potent inflammatory form of cell death, pyroptosis, which would significantly exacerbate this inflammatory condition. A number of alternative roles for the non-canonical inflammatory caspases are also emerging, including roles in actin remodelling and crosstalk with other cell signalling pathways, such as the STAT1 pathway (Papoff et al., 2018; Caution et al., 2019; Flood et al., 2019). In the context of IBD, these pyroptosis-independent functions could contribute to the disruption of epithelial intestinal barriers and the epigenetic enhancement of IBD inflammation, respectively (Lechuga and Ivanov, 2021; Yu et al., 2021). Evidence to date therefore suggests that further investigation into the complex molecular mechanisms of caspase-4 and -5 regulation, activation and signalling has the potential to provide new strategies for therapeutic intervention in inflammatory bowel diseases.
